# Applications of diffusion tensor imaging integrated with neuronavigation to prevent visual damage during tumor resection in the optic radiation area

**DOI:** 10.3389/fonc.2022.955418

**Published:** 2022-08-16

**Authors:** Jianwei Shi, Dafeng Lu, Ruihan Pan, Hairong Chen, Hong Teng, Yang Xu, Fuduo Bo, Qi Zhou, Yansong Zhang

**Affiliations:** ^1^ Department of Neurosurgery, The Affiliated Brain Hospital of Nanjing Medical University, Nanjing, China; ^2^ School of Public Health, Nanjing Medical University, Nanjing, China; ^3^ Department of Neurosurgery, First Affliated Hospital, Zhejiang Chinese Medical University, Hangzhou, China; ^4^ Department of Geriatrics , The Affiliated Brain Hospital of Nanjing Medical University, Nanjing, China

**Keywords:** neuronavigation, diffusion tensor imaging, tumor resection, optic radiation, visual function

## Abstract

**Background:**

Intracranial tumors involving the temporo-occipital lobe often compress or destroy the optic radiation (OpR), resulting in decreased visual function. The aim of this study is to explore the value of diffusion tensor imaging (DTI) tractography integrated with neuronavigation to prevent visual damage when resecting tumors involving the OpR and find potential factors affecting patients’ visual function and quality of life (QOL).

**Methods:**

Our study is a cross-sectional study that included 28 patients with intracranial tumors in close morphological relationship with the OpR recruited between January 2020 and February 2022. The surgical incision and approach were preoperatively designed and adjusted according to the DTI tractography results and visual function scores. All patients underwent examinations of visual acuity (VA) and visual field index (VFI) and completed visual function and QOL scales at admission and 2 months after discharge. Logistic regression and linear regression analysis were conducted to evaluate clinical factors potentially affecting pre/postoperative OpR morphology, VA, VFI, visual function, and QOL.

**Results:**

Lesion size was the main factor found to affect visual function (β = -0.74, 95%CI: -1.12~-0.36, P = 0.05), VA (left: β = -0.11, 95%CI: -0.14~-0.08, P < 0.001; right: β = -0.15, 95%CI: -0.17~-0.13, P < 0.001), and VFI (left: β = -0.11, 95%CI: -0.14~-0.08, P < 0.001; right: β = -0.14, 95%CI: -0.16~-0.12, P < 0.001). Lesion size, edema, and involvement of the lateral ventricle temporal horn were factors affecting OpR morphology and QOL. The 28 patients showed significantly improved VA, VFI, visual function, and QOL results (P < 0.05) 2 months after discharge.

**Conclusions:**

Combining DTI of OpR mapping and microscopic-based neuronavigation aided precise mapping and thus preservation of visual function in patients undergoing tumor resection. Potential clinical factors affecting patients’ visual function and QOL scores were identified which are useful for assessing a patient’s condition and predicting prognosis.

## Introduction

The optic radiation (OpR) begins in the lateral geniculate body and occupies the temporal and parietal lobes in the striatum. Many brain lesions can involve the OpR and cause decreased visual function, visual acuity (VA), and visual field index (VFI). As the most anterior part of the OpR and an important anatomical location in neurosurgery, Meyer’s loop projects forward across the superior aspect of the anterior tip of the lateral ventricle’s temporal horn. During tumor resection, the protection of Meyer’s loop remains challenging, which is responsible for patients’ prost-operative visual function and quality of life (QOL) (1). Notably, tractography of the OpR has been shown to limit surgical damage to visual function during tumor resection involving the OpR area (2).

The OpR cannot be distinguished using clinical magnetic resonance imaging (MRI) sequences. Diffusion tensor imaging (DTI) is an advanced MRI technique to evaluate microstructural changes in the brain using water diffusion as the MR contrast and collecting diffusion-weighted (DW) MR images (3). At present, DTI research mainly focuses on tracking neural fiber pathways in the central nervous system. Notably, DTI tractography enables the delineation of the OpR, which can be used to guide the surgeon to prevent visual damage during intracranial tumor resection planning. This surgical strategy can assist with intra-operative planning to decrease the chances of damaging the functional nerves (e.g., OpR, facial nerve, vestibular nerve) and improve patients’ QOL following surgery (4–6). DTI integrated with neuronavigation can allow neurosurgeons to evaluate the extent of tumor resection and modify the surgical strategy in real-time, resulting in better surgical results and lowering risks (4, 7).

Intracranial tumor patients with visual impairment are constantly facing challenges in achieving an independent and productive life, so prevention of visual damage during resection is of great value. The primary aim of this study was to examine the potential use of DTI integrated with neuronavigation in surgical planning and intraoperative guidance to predict the anatomical location of the visual pathways and preserve visual function during tumor resection. The secondary aims were to identify clinical factors that may affect OpR morphology, VA, VFI, visual function, and QOL.

## Material and methods

### Subjects

Twenty-eight patients (13 males; age range: 12–78 years; mean ± standard deviation (SD): 53 ± 21 years) with intracranial tumors touching (the minimum distance between OpR and tumor ≤ 1 cm), pushing, wrapping, or invading the OpR were prospectively recruited to participate in this single-center cross-sectional study at Nanjing Brain Hospital, Nanjing, China, between January 2020 and February 2022. The exclusion criteria were: (1) patients who received craniotomy or radiotherapy before admission; (2) patients with independent ophthalmic diseases or anterior visual pathway dysfunction; (3) patients who refused or did not complete follow-up; and (4) patients who could not cooperate with or complete DTI during MRI scanning.

Demographic data, tumor size, the presence/absence of edema, involvement of the lateral ventricle temporal horn, the extent of resection (EOR), VA, VFI, visual function, and QOL data were collected for all patients. This study was approved by the Nanjing Brain Hospital Ethics Committee and informed written consent was obtained from all patients.

### Visual function evaluation

All patients underwent examination of binocular VA and VFI and completed the visual function and QOL scales at admission and 2 months after discharge. The QOL and visual function scales, which were developed by the World Health Organization and American Eye Institute for developing countries, are provided in **Supplementary Table 1, 2** (8, 9).

### Imaging and fiber tracking

Patients underwent structural MRI and DTI imaging on a 3.0 Tesla MRI scanner (Siemens Magnetom TIM Trio, Erlangen, Germany) before surgery. The lesion size was represented by the product of the maximum diameter of the axial, sagittal, and coronal T1-weighted volumetric acquisition sequence. While including extra- and intra- axial tumors, the minimum distance between the tumor and OpR was measured as well. DTI data were acquired by interlaced echo-planar imaging (IEPI) with 2.0 mm isotropic voxels and 32 directions with a *b* value of 1,000 s/mm^2^ (repetition time (TR) = 6100 ms, echo time (TE) = 106 ms, section thickness = 1.0 mm). All patients underwent a contrast-enhanced MRI one day after surgery, and preoperative MRI was used to evaluate EOR.

The collected MRI data were transferred to a Stealth Station navigation system (Medtronic-Sofamor Danek, USA) and StealthViz ^®^ (Medtronic-Sofamor Danek, USA) software was used to process fiber tractography (fractional anisotropy = 0.2, apparent diffusion coefficient = 0.1). The fiber diameter was 0.2 mm (10). The maximal angle was 45°. After preprocessing (and spatial smoothing, when applied), the lateral geniculate body and occipital cortex, including the calcarine sulcus, were divided into regions of interest (ROIs). The size and direction of the ROIs were adjusted until we found clear fiber bundles passing through the defined ROI using knowledge of each patient’s anatomy (Carried out by JS & RP, and then censored by HT). Bundles that could contaminate the optic tracts were eliminated. An OpR morphology score was generated for each patient ranging from 1 to 4, with a higher score indicating greater OpR damage (Carried out by JS & RP, and then censored by YZ) (11).

### 3D reconstruction of OpR

Cranial^®^ software implemented on a Stealth Station (Medtronic-Sofamor Danek, USA) was used to construct 3D models of the OpR and lesions, and neuronavigation plans were subsequently made. The surgical approach was designed according to the 3D positional relationship between the OpR and the lesion.

### Intraoperative neuronavigation and surgery

Targeting was implemented through the guidance of the fiber bundle fusion neuronavigation system. The entry and target points were marked according to the preoperatively designed surgical approach. The distance between the operating position and the OpR was judged in real-time to avoid damage to the OpR. Less aggressive resections given DTI guidance are beneficial. For tumors with complicated locations or invading the OpR, a navigation-guided needle was used to puncture to the boundary between the OpR and the tumor after opening the dura mater. A small piece of methylene blue-colored gelatin sponge was pushed in to help locate the OpR. After reaching the blue-stained area, special attention must be paid. We gently separated and pulled the lesion and peritumoral tissue to the affected side and protected the identified OpR to prevent postoperative visual damage. The use of bipolar electrocoagulation should be reduced and switched to absorbable hemostatic agents such as Surgicel or physical compression hemostasis. If final pathologic results suggest malignant tumors, follow-up radiotherapy and chemotherapy should be given.

The EOR corresponds to the percentage of volume resected with respect to the preoperative volume and was classified as follows: gross total resection, EOR = 100%; near total resection, 95% ≤ EOR < 100%; subtotal resection, 80% ≤ EOR < 95%; partial resection < 80% (12, 13).

### Statistical analysis

The paired Student’s *t*-test was used to evaluate the efficacy of surgery by comparing the QOL, visual function, VA, and VFI values between admission and 2 months after discharge with normal distribution. If these variables do not normally distribute, the paired Wilcoxon signed-rank test will be applied. Logistic regression analysis was performed for binary variables. Linear regression was used for continuous variables with a normal distribution and variables that did not conform to the normal distribution were converted to binary variables and then analyzed using logistic regression. Multivariate logistic regression adopting the backward elimination technique was conducted to evaluate potential risk factors associated with unfavorable results of OpR morphology, VA, VFI, visual function, and QOL. Statistical analyses were performed using R software (version 4.04). All tests were two-tailed and results were considered statistically significant at the 0.05 level unless otherwise specified.

## Results

### Patient clinical data

**Figure 1:**
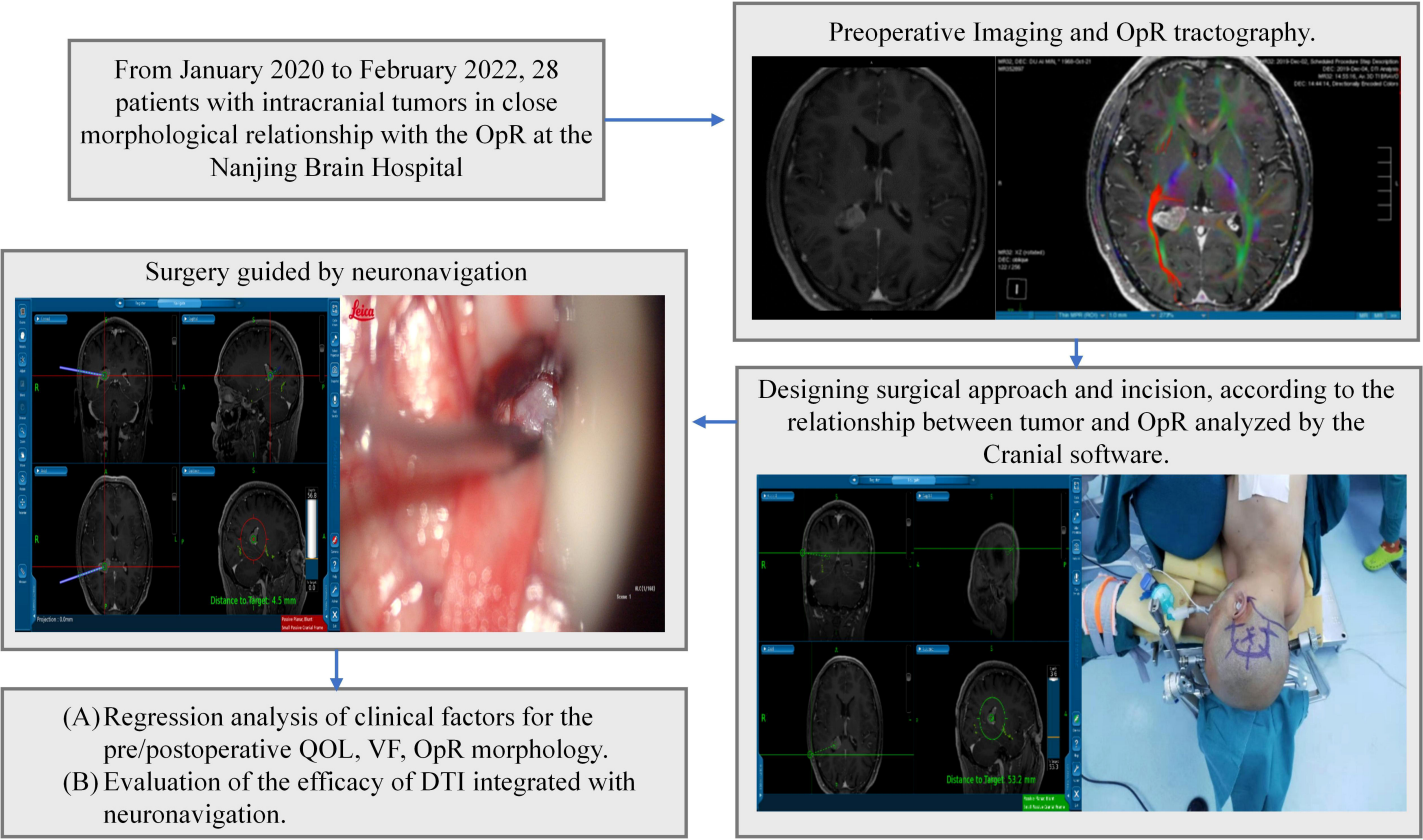
Study design. Intraoperative images were acquired following initial dissection and at the end of surgery. OpR, optic radiation, DTI; diffusion tensor imaging, VF; visual function; QOL, quality of life.

Patient demographics and clinical information are listed in **Table 1**. The results of the visual exam and QOF of 28 patients are presented in **Table 2**. The most frequent complaint on admission was headache and dizziness (n=10, 35.7%), followed by visual deterioration (n=9, 32.1%), seizures (n=6, 21.4%), aphasia (n=2, 7.1%), and numbness (n=1, 3.5%). The mean duration from admission to discharge was 19.8 ± 2.9 days. The lesions included 14 gliomas, 4 meningiomas, 4 cavernous hemangiomas, 3 metastases, and 3 lymphomas. A flowchart of the study design is illustrated in **Figure 1**. The detailed regression analysis results related to patient characteristics are listed in **Supplementary Table 3**.

**Table 1:** Patient demographics and clinical information.

No.	Sex	Age (years)	Position	Lesion Size	Pathological diagnosis	Edema	Invasion of the temporal horn	OpR Score (s)	Lesion Excision
1	F	21	Right temporal lobe	2.0*2.0*1.5cm	Ganglioglioma	N	Y	1	Gross total resection
2	F	63	Right temporal lobe	5.0*5.0*4.0cm	Meningioma	N	N	2	Gross total resection
3	M	68	Left temporal lobe	2.0*1.5*1.5cm	Glioblastoma	N	N	1	Gross total resection
4	M	78	Left temporal lobe	4.7*3.7*3.9cm	Glioblastoma	Y	Y	4	Near total resection
5	F	74	Left temporal lobe	4.0*3.1*2.1cm	Brain metastasis	Y	N	2	Gross total resection
6	F	65	Left parietal occipital falx	3.5*3.0*3.2cm	Meningioma	N	N	2	Gross total resection
7	M	54	Left temporal lobe	2.0*1.4*1.4cm	Pilocytic astrocytoma	N	N	2	Gross total resection
8	M	61	Left parietal occipital lobe	3.5*3.1*2.5cm	Brain metastasis	Y	N	3	Gross total resection
9	M	70	Right temporal lobe	4.6*2.9*2.2cm	Lymphoma	Y	N	3	Partial resection
10	F	60	Right occipital canopy	2.4*1.5*1.6cm	Meningioma	N	N	1	Gross total resection
11	M	53	Right temporo-parietal occipital lobe	4.8*5.0*3.8cm	Glioblastoma	Y	Y	4	Near total resection
12	F	54	Left temporal insula	2.2*1.7*1.5cm	Glioblastoma	Y	Y	3	Gross total resection
13	M	71	Right temporal lobe	5.8*3.4*3.6cm	Brain metastasis	Y	Y	4	Subtotal resection
14	F	21	Right temporal lobe	1.2*0.8*0.6cm	Cavernous hemangioma	N	N	1	Gross total resection
15	M	67	Left temporo-parietal occipital lobe	5.6*4.5*3.0cm	Glioblastoma	Y	Y	4	Near total resection
16	F	12	Right parietal occipital lobe	3.0*2.0*2.2cm	Astrocytoma	Y	N	2	Gross total resection
17	M	12	Left temporal lobe	1.8*1.2*1.0cm	Cavernous hemangioma	Y	N	1	Gross total resection
18	F	62	Left ventricle and temporal horn	3.0*2.8*2.0cm	Anaplastic astrocytoma	Y	Y	3	Subtotal resection
19	F	15	Right temporal lobe	4.7*3.0*4.5cm	Cavernous hemangioma	Y	N	3	Gross total resection
20	M	56	Left temporal lobe	1.5*1.5*1.2cm	Ganglioglioma	N	Y	1	Gross total resection
21	M	61	Left occipital lobe	1.8*2.2*2.8cm	Cavernous hemangioma	N	N	2	Gross total resection
22	F	20	Left temporal lobe	2.9*2.0*1.7cm	Diffuse glioma	N	N	2	Gross total resection
23	F	72	Left ventricle and temporal horn	2.6*1.5*1.3cm	Lymphoma	Y	Y	3	Partial resection
24	M	63	Left temporal insula	4.5*4.6*4.3cm	Anaplastic Glioma	Y	Y	3	Gross total resection
25	M	49	Left temporal lobe	5.0*3.7*4.5cm	Glioblastoma	Y	Y	4	Gross total resection
26	F	68	Left temporal insula	4.0*3.0*2.6cm	Glioblastoma	Y	Y	3	Near total resection
27	F	76	Left temporal lobe	5.8*4.2*3.0cm	Meningioma	Y	N	2	Gross total resection
28	F	41	Left temporo-parietal insula	7.0*5.0*4.8cm	Diffuse glioma	Y	Y	3	Near total resection

(F, female; M, male; Y, yes; N, no; OpR, optic radiation).

*means ×, which is a multiplication sign.

**Table 2:** The results of pre-operative visual exam and QOF compared to post-operative of 28 patients.

No.	Pre-QOL	Post-QOL	Pre-VF	Post-VF	Pre-VA (L)	Post-VA (L)	Pre-VA (R)	Post-VA (R)	Pre-VFI (L)	Post-VFI (L)	Pre-VFI (R)	Post-VFI (R)
1	100.00	100.00	85.67	87.50	0.60	0.60	0.80	0.80	0.99	0.99	0.96	0.96
2	85.43	85.43	54.63	60.18	0.20	0.30	0.50	0.50	0.32	0.33	0.24	0.25
3	100.00	100.00	88.90	88.90	0.80	0.80	1.00	1.00	0.84	0.84	0.91	0.92
4	76.40	76.42	57.42	62.97	0.10	0.10	0.30	0.30	0.21	0.22	0.32	0.34
5	76.40	76.4.0	73.15	78.70	0.50	0.60	0.80	0.80	0.54	0.54	0.47	0.49
6	85.43	88.20	67.60	78.70	0.20	0.20	0.60	0.60	0.47	0.50	0.48	0.48
7	97.23	97.23	87.05	88.90	0.80	0.90	1.00	1.00	0.87	0.87	0.82	0.84
8	86.10	86.10	73.15	73.15	0.50	0.60	0.70	0.70	0.52	0.52	0.49	0.48
9	86.10	86.10	84.27	84.27	0.70	0.70	0.40	0.50	0.36	0.38	0.41	0.42
10	100.00	100.00	88.90	88.90	1.00	1.00	0.80	0.80	0.85	0.88	0.90	0.90
11	70.85	72.93	59.73	65.28	0.40	0.60	0.20	0.30	0.30	0.30	0.25	0.30
12	94.45	97.23	87.50	87.50	0.60	0.80	1.00	1.00	0.77	0.79	0.81	0.83
13	77.78	77.80	70.38	75.93	0.60	0.60	0.20	0.30	0.32	0.32	0.29	0.30
14	100.00	100.00	88.89	88.89	1.20	1.20	1.00	1.00	0.89	0.90	0.91	0.91
15	65.30	65.30	56.03	61.58	0.20	0.20	0.40	0.50	0.36	0.39	0.32	0.32
16	100.00	100.00	88.89	88.89	1.00	1.00	0.80	0.90	0.77	0.80	0.71	0.74
17	100.00	100.00	88.89	88.89	1.00	1.00	1.20	1.20	0.92	0.93	0.95	0.97
18	61.13	63.91	78.70	78.70	0.50	0.60	0.80	0.80	0.54	0.54	0.60	0.61
19	90.98	90.98	78.70	78.70	0.60	0.60	0.40	0.50	0.45	0.48	0.35	0.40
20	97.23	97.36	88.89	88.89	1.00	1.00	1.20	1.20	0.95	0.95	0.94	0.96
21	100.00	100.00	88.89	88.89	0.80	0.80	1.00	1.00	0.95	0.95	0.96	0.96
22	100.00	100.00	87.50	88.89	0.80	0.80	1.00	1.00	0.91	0.93	0.95	0.94
23	81.25	81.25	78.70	84.25	0.60	0.60	0.80	0.80	0.82	0.85	0.79	0.82
24	73.63	76.40	75.93	77.78	0.20	0.30	0.60	0.60	0.26	0.29	0.29	0.30
25	77.78	77.78	73.15	75.93	0.40	0.40	0.50	0.50	0.42	0.43	0.39	0.39
26	77.78	78.70	78.70	78.70	0.30	0.30	0.50	0.60	0.56	0.58	0.56	0.56
27	86.10	88.90	84.27	87.05	0.50	0.60	0.70	0.80	0.67	0.68	0.59	0.60
28	86.10	90.98	78.70	78.70	0.30	0.50	0.60	0.70	0.48	0.49	0.48	0.48

(QOL; quality of life, VF; visual function, VA; visual acuity, VFI; visual field index, L; left, R; right, Pre; preoperative/at admission, Post; postoperative/2 months after admission).

### OpR tracking

All 28 patients had normal OpR morphology on the unaffected side. Regarding OpR morphology on the affected side, 6 cases (grade 1, 21.4%) were morphologically normal, 8 cases (grade 2, 28.6%) were squeezed, 9 cases (grade 3, 32.1%) were deformed or partially destroyed, and 5 cases (grade 4, 17.9%) were interrupted. The mean OpR morphology score of the affected side was 2.5 ± 1.0 points. Lesion size (odds ratio, OR = 0.75, 95% confidence interval, 95% CI: 0.634–0.886, P = 0.003), edema (OR = 0.486, 95%CI: 0.281–0.842, P = 0.017), and involvement of the lateral ventricle temporal horn (OR = 0.551, 95% CI: 0.349–0.87, P = 0.018) were the main clinical factors associated with OpR morphological damage (**Supplementary Table 3**). The minimum distance between the tumor and OpR was only able to be measured in patients with an OpR score of one and was 0.3 cm, 0.7 cm, 1.0 cm, 0.5 cm, 0.3 cm, and 0.4 cm in order.

### Analysis of preoperative VA, VFI, visual function, and QOL

Multivariate linear regression analysis was performed for the preoperative variables. Lesion size was identified as more likely factor affecting preoperative visual function (β = -0.74, 95%CI: -1.12~-0.36, P = 0.05), VA (left: β = -0.11, 95%CI: -0.14~-0.08, P < 0.001; right: β = -0.15, 95%CI: -0.17~-0.13, P < 0.001), and VFI (left: β = -0.11, 95%CI: -0.14~-0.08, P < 0.001; right: β = -0.14, 95%CI: -0.16~-0.12, P < 0.001). Advanced age and edema were potential factors for damaged VA and VFI. Advanced age (β = -5.33, 95%CI: -7.01~-3.65, P = 0.004), edema (β = -7.46, 95%CI: -10.83~-4.09, P = 0.037), and involvement of the lateral ventricle temporal horn (β = -7.71, 95%CI: -10.50~-4.92, P = 0.011) were the main risk factors affecting preoperative QOL (**Supplementary Table 3**).

### Analysis of postoperative VA, VFI, visual function, and QOL

Multivariate logistic regression analysis was performed for the postoperative variables. We did not find a significant association of VA or VFI with sex, age, tumor size, edema, invasion into the temporal horn, the extent of tumor resection, or OpR morphology. In the univariate regression analysis, tumor size (OR = 4.12, 95%CI: 1.75~16.36, P = 0.01) and edema (OR = 1.65, 95%CI: 1.20~2.27, P = 0.01) were significantly related to right VA (β > 0). However, this result does not mean that larger tumor size and more serious edema result in better VA. Preoperative univariate regression analysis showed that right VA was also significantly associated with tumor size (β = -0.14, 95%CI: -0.16~-0.12, P < 0.001) and edema (β = -0.30, 95%CI: -0.39~-0.21, P = 0.002). Taken together, these results suggest that the impact of tumor size and edema on patients’ VA may be recovered after surgery. OpR morphology was the main factor affecting postoperative visual function (OR = 0.203, 95% CI: 0.036–0.731, P = 0.033) while involvement of the lateral ventricle temporal horn (OR = 17.542, 95% CI: 2.678–206.021, P = 0.007) was the main clinical factor affecting postoperative QOL (**Supplementary Table 3**).

### Intraoperative neuronavigation and surgery

**Figure 2:**
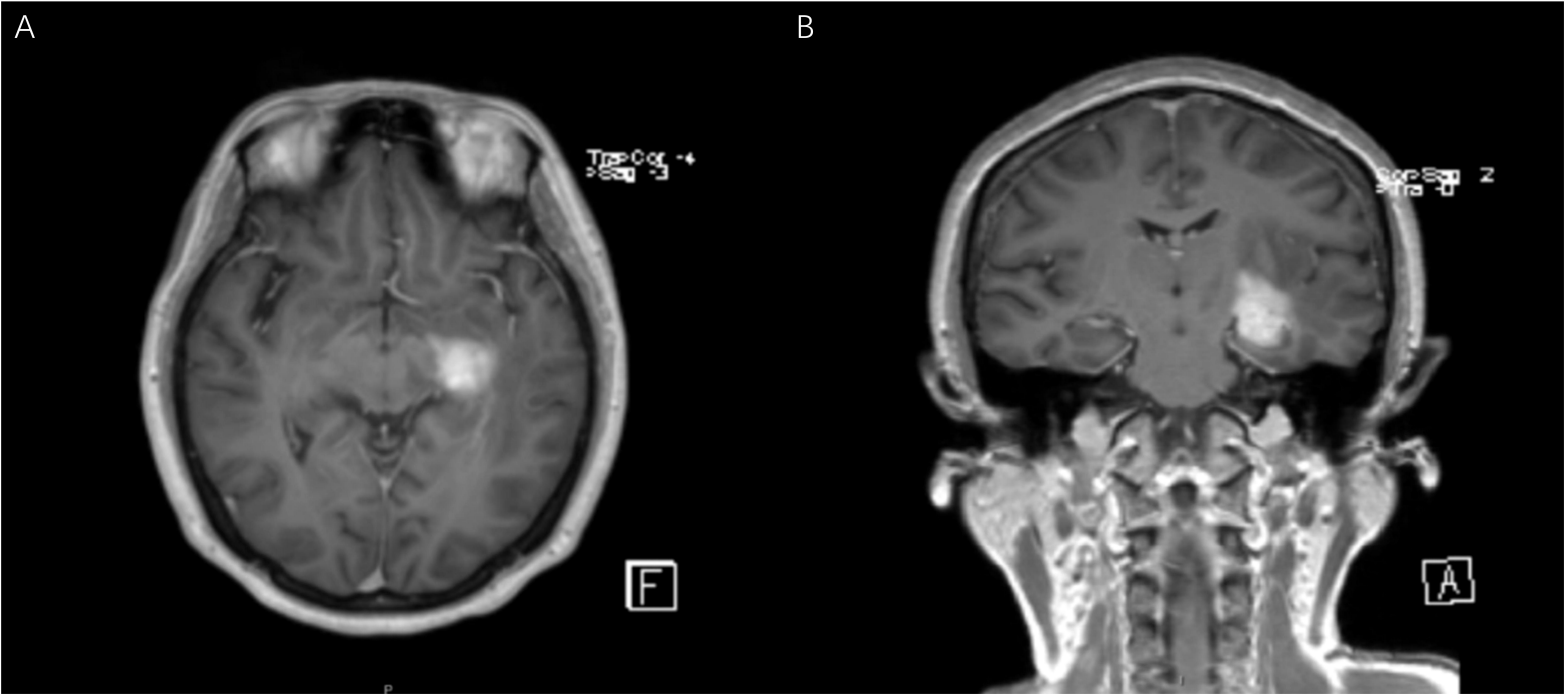
Patient No.12 is a 54-year-old female with a main complaint of dizziness. The coronal **(A)** and axial **(B)** MR T1 weighted images showed a 2.2×1.7×1.5cm abnormal signal shadow in the left temporal insula. The initial diagnosis was glioma.

**Figure 3:**
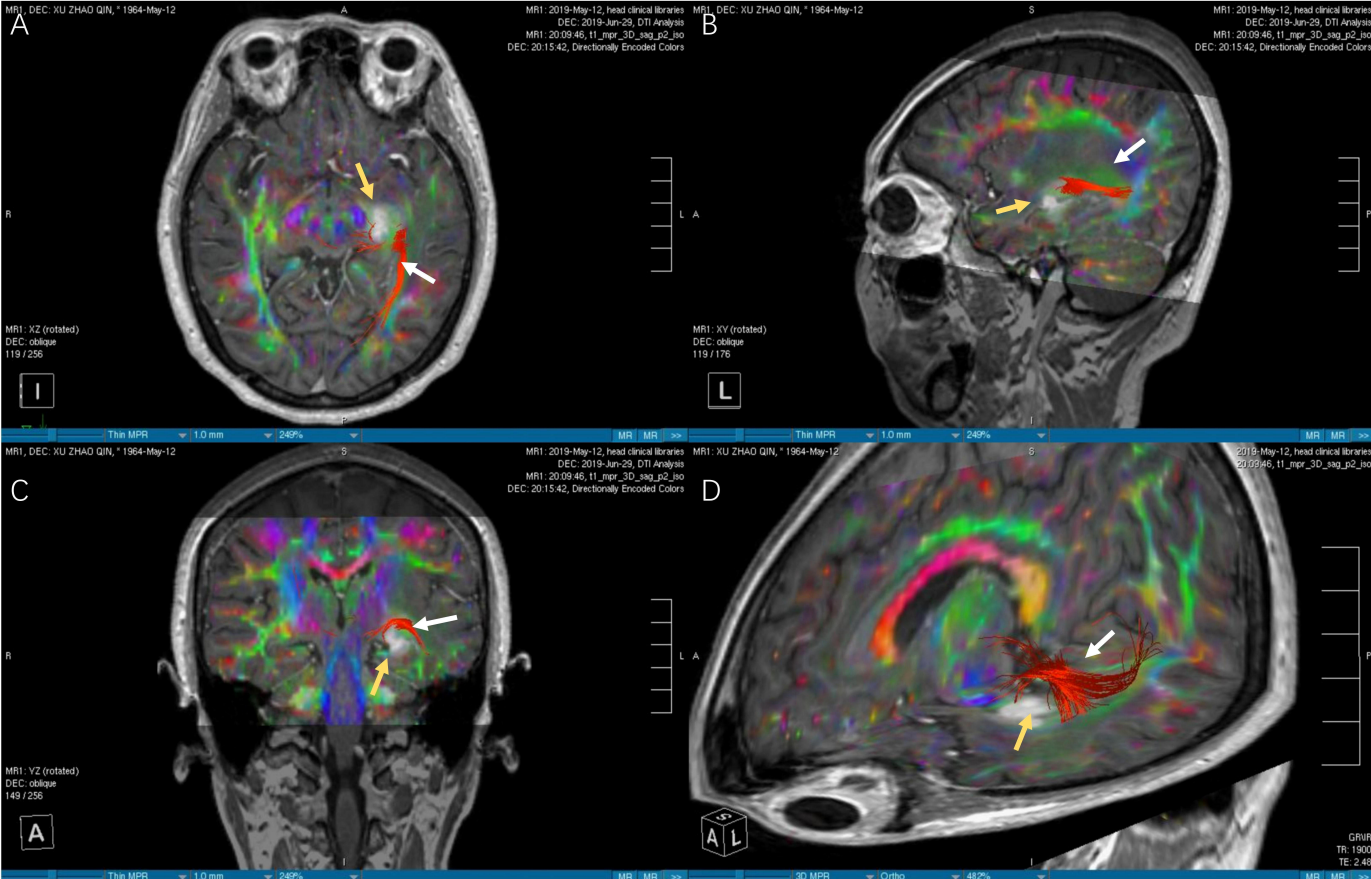
The images of DTI tractography of patient No.12 (OpR morphology score = 3). The DTI data was imported into the StealthViz ^®^ software for neuronavigation. The lateral geniculate body and the rectangular sulcus were set as regions of interest to reconstruct the OpR on the affected side. **(A)** The axial image showed partial disruption of left OpR. **(B)** In sagittal view, the OpR on the affected side can be seen behind and above the tumor. **(C)** The coronal image showed that the tumor pushed the starting position of the OpR upward. **(D)** The OpR was partially interrupted (OpR morphology score=3) and was located above and behind the tumor, based on 3D reconstruction. Therefore, the inferior temporal gyrus approach was applied to avoid OpR. The white arrow indicates the OpR and the yellow arrow indicates the tumor. DTI, diffusion tensor imaging, OpR, optic radiation.

**Figure 4:**
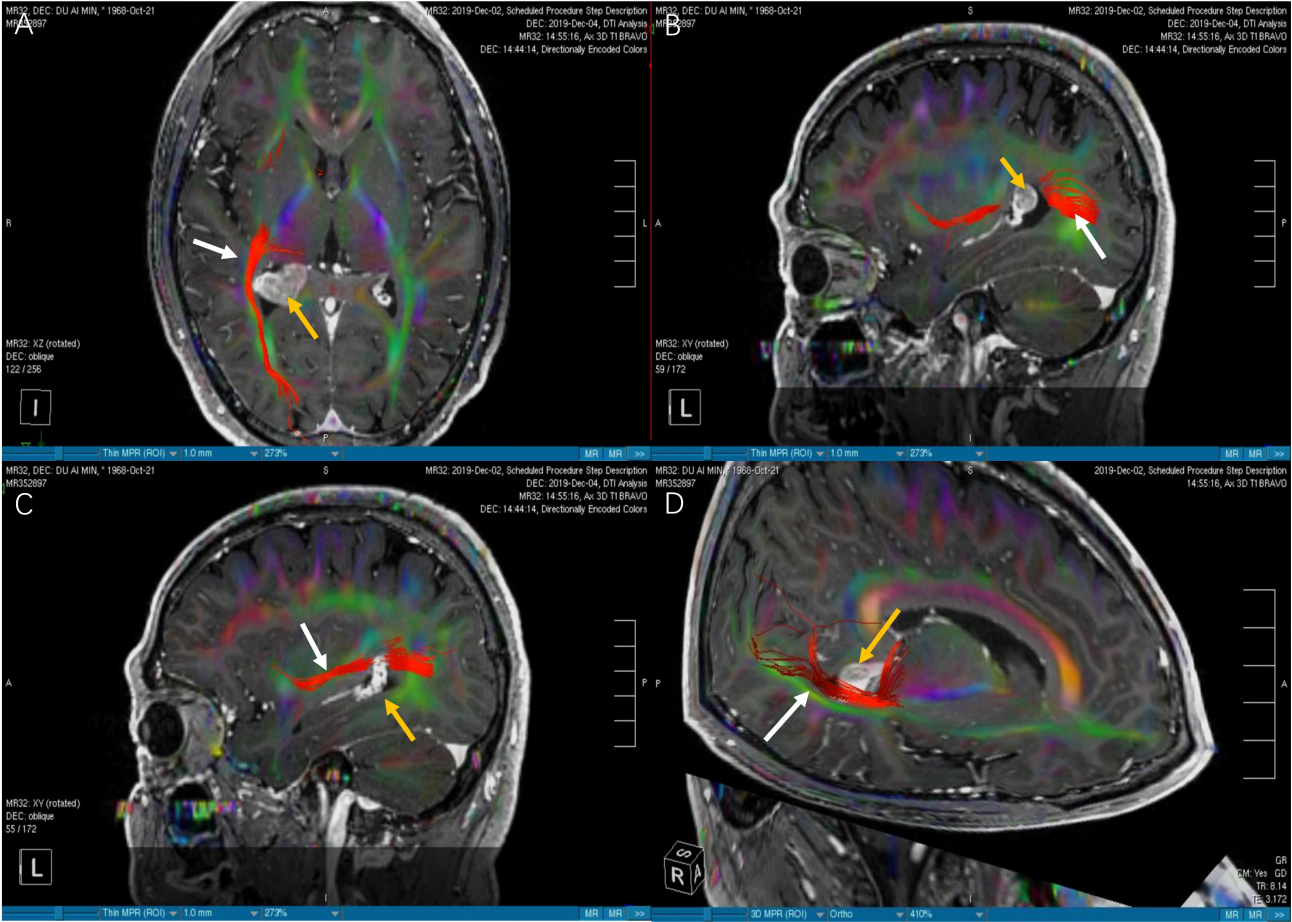
The images of DTI tractography of patient No.6 (OpR morphology score = 2). The DTI data was imported into the StealthViz ^®^ software for neuronavigation. The lateral geniculate body and the rectangular sulcus were set as regions of interest to reconstruct the OpR on the affected side. **(A)** Compared with **Figure 3A**, the axial image showed that the right OpR was normal and healthy. **(B)** In sagittal view, the OpR on the affected side was located at the edge of the lateral ventricular triangle. **(C)** We found that the OpR appeared to be on the lateral of the tumor. But it had the possiblity that the OpR was partially or completely interrupted. **(D)** The 3D reconstruction confirmed that the OpR was just slightly pushed so a OpR morphology score of 2 was given. The white arrow indicates the OpR and the yellow arrow indicates the tumor. DTI; diffusion tensor imaging, OpR; optic radiation.

**Figure 5:**
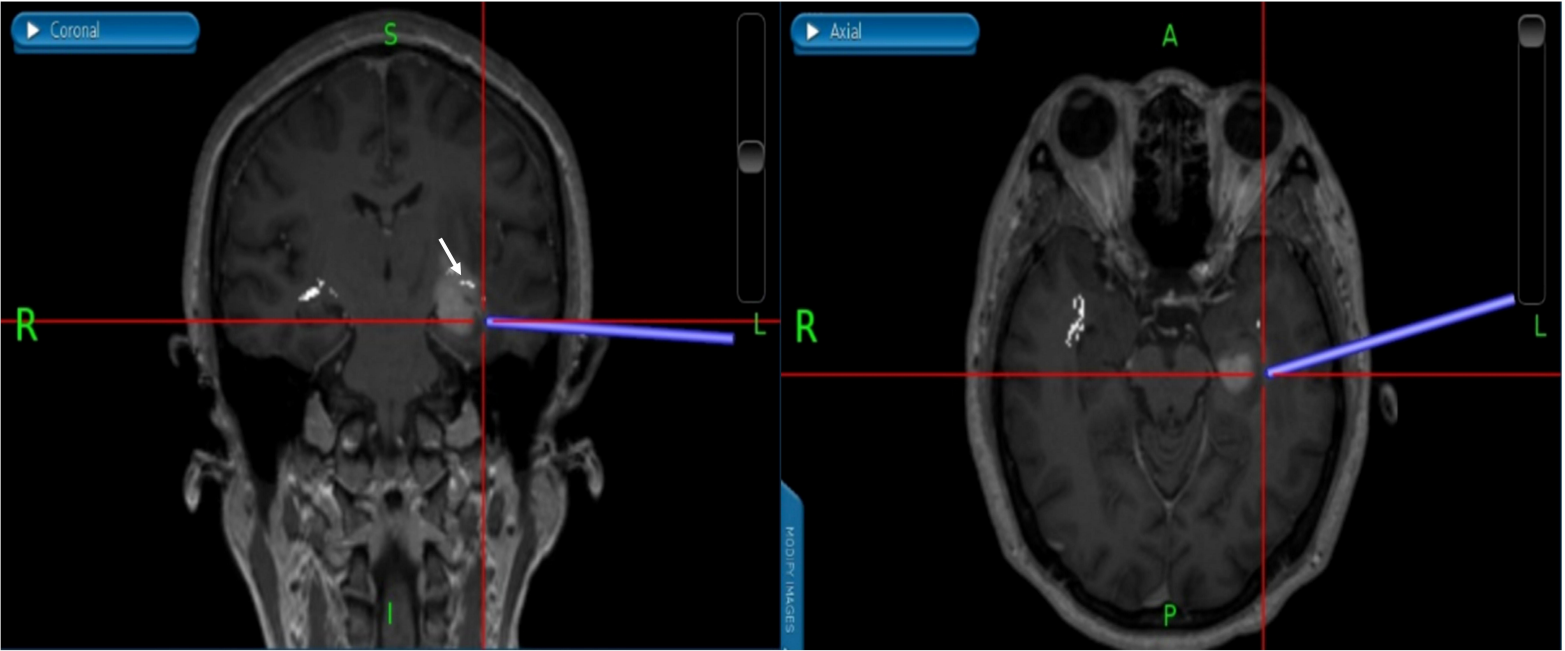
(Patient No.12) According to the relationship between tumor and OpR, the surgical approach is adjusted to the inferior temporal gyrus approach. We successfully reached the tumor under the guidance of neuronavigation and avoided OpR damage. The white part indicated by the white arrow is the OpR. OpR, optic radiation.

**Figure 6:**
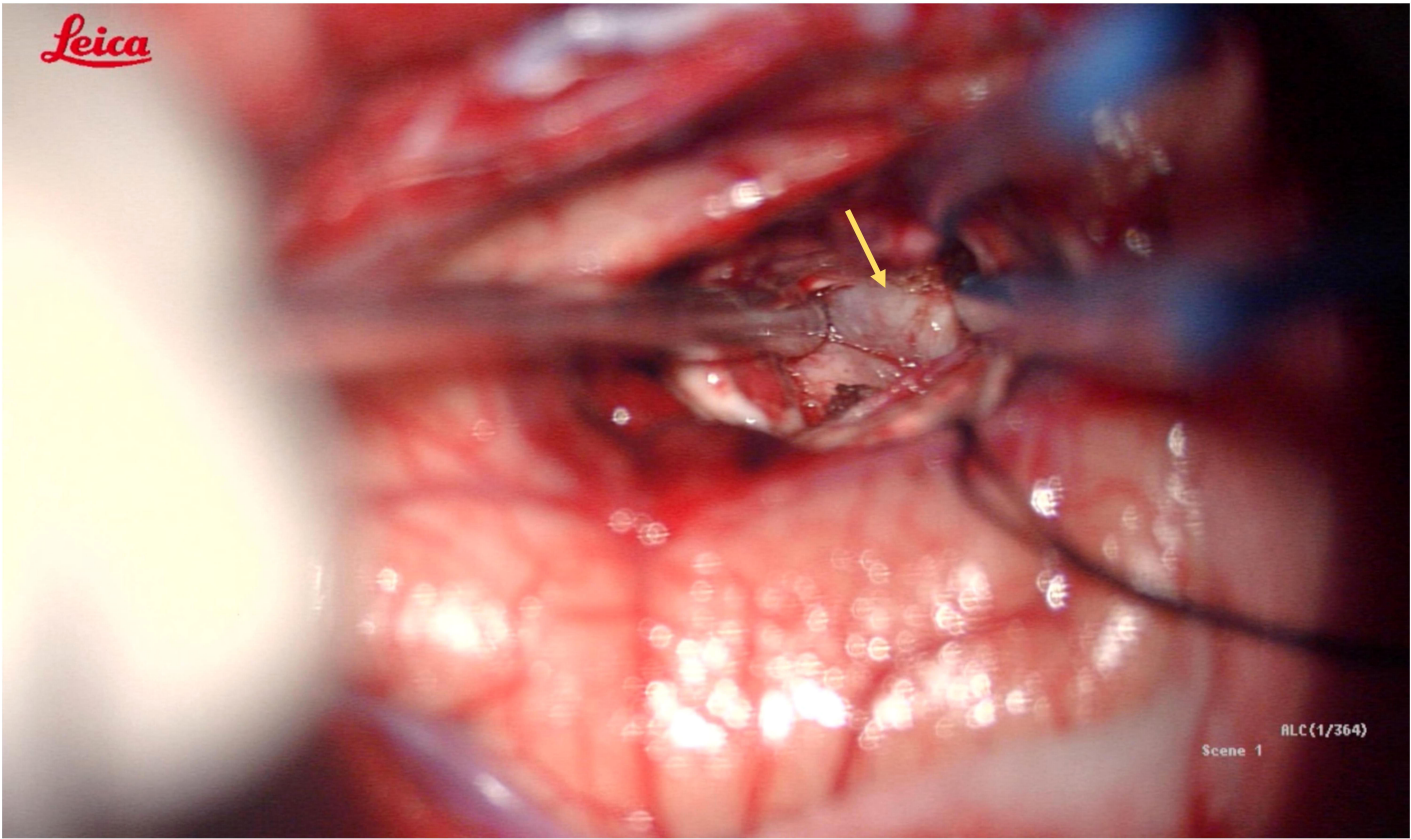
(Patient No.12) During the operation, a gray-white tumor with tough texture, unclear boundary and normal blood supply was seen. The rapid pathology suggested an anaplastic glioma (WHO III). The yellow arrow indicates the tumor.

**Figure 7:**
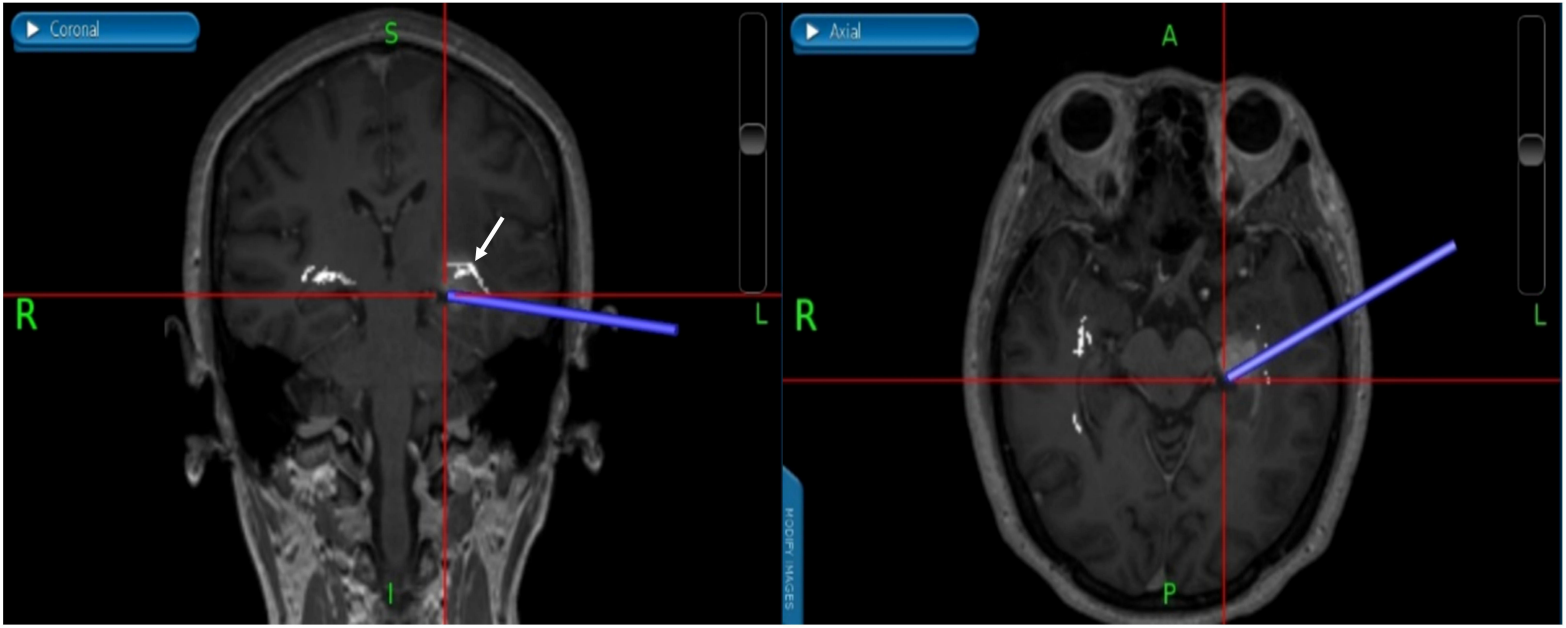
(Patient No.12) We used neuronavigation to avoid damage to the brain stem and the real time neuronavigation imaging showed that gross total resection was performed without any injuries of the OpR. The white arrow indicates the OpR. (OpR: optic radiation).

**Figure 8:**
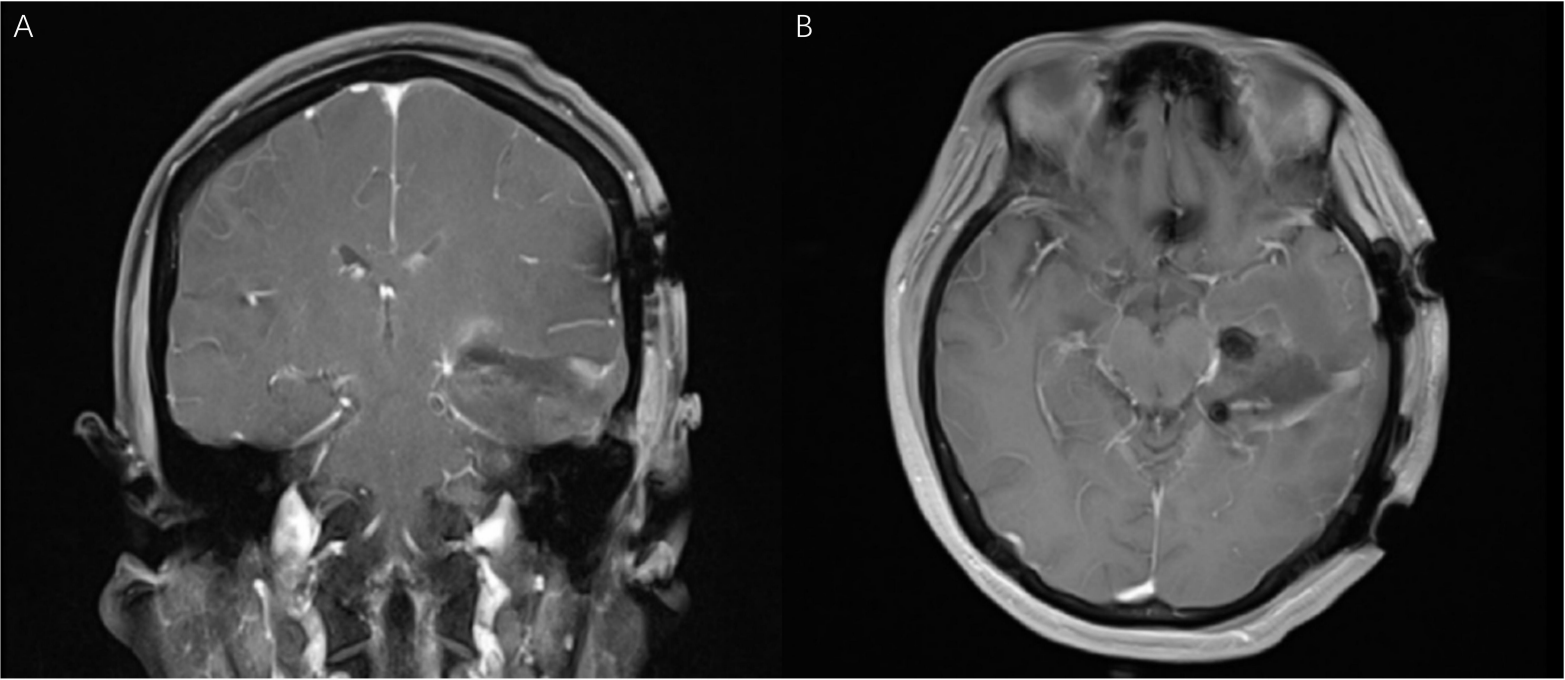
(Patient No.12) The coronal **(A)** and axial **(B)** MR T1 weighted images (one day after operation) confirmed the gross total resection of the tumor. The visual functions were well protected.

According to the preoperative DTI results, 16 patients underwent a surgical approach designed by traditional experience (11 cases employed the trans-cortical approach, 2 cases employed the subtemporal approach, 1 case employed the trans-sylvian approach and 2 cases employed the occipital-transtentorial approach/Poppen approach) and 12 patients underwent a readjusted trans-cortical approach according to the OpR position reconstructed by DTI to avoid damage to the OpR (**Figures 2**–**8**). Nineteen cases (68%, 7 gliomas, 4 meningiomas, 4 cavernous hemangiomas, 2 metastases, and 2 lymphomas) underwent gross total resection, 5 cases (18%; 5 gliomas) underwent near total resection, 2 cases (7%; 1 anaplastic astrocytoma, 1 metastasis) underwent subtotal resection, and 2 cases (7%; 2 lymphomas) underwent partial resection.

### Efficacy and prognosis

Since the variables did not follow a normal distribution, the paired samples Wilcoxon signed rank test was applied for analysis. Although 7 patients experienced a transient decrease in visual function after surgery caused by brain tissue edema, no patients had worsening VA or VFI two months after discharge. Five patients developed focal impaired awareness autonomic/clonic seizures postoperatively (14). No other discomfort or complications were reported by patients. Two months after discharge, the VA (left, P = 0.002; right, P = 0.002) and VFI (left, P < 0.001; right, P < 0.001) of the included patients had a statistically significant difference relative to the time of admission (**Table 2**; **Supplementary Table 4**). Significant differences in visual function (P = 0.001) and QOL (P = 0.002) scores between admission and 2 months after discharge were found for all patients (**Supplementary Table 4**).

## Discussion

### Main findings and interpretation

Accurate intraoperative localization of the OpR using tractography has been shown to be effective in neurosurgery (15). At present, three methods can be applied to study the OpR: (i) determining the relationship between visual field deficits and resected tissue by evaluating the postoperative VA of patients with temporal lobe lesions, which is commonly used in studies of anterior temporal lobectomy for temporal lobe epilepsy (1, 5, 16); (ii) performing anatomical studies using a special fiber separation technique (Klingler’s fiber dissection technique) (17); and (iii) DTI and diffusion tensor tractography (DTT) based on DTI (7). StealthViz^®^ is a convenient software application for quick and easy fiber tract imaging that is helpful for neurosurgical planning and navigation (18). Based on anatomy, we adopted the tracking method described by Bertani (19) and Sherbondy (20) to establish ROIs and display the OpR’s course. Our results suggest that DTI integrated with neuronavigation is useful and reliable for designing surgical strategies to avoid OpR injury during tumor resection. Potential factors affecting patients’ visual function and QOL scores were identified simultaneously.

The most common postoperative complications of surgical treatment for temporo-occipital lobe lesions are decreased VA/VFI, epilepsy, and aphasia (21, 22). Declined visual function and QOL after surgery are very common (23, 24). Prior studies have reported that more than 50% of patients with epilepsy develop visual field deficits after anterior temporal lobectomy (16, 25). There are two main reasons for this risk: (i) direct damage to the OpR and visual cortex through the transcortical approach; (ii) intraoperative damage to arteries supplying the OpR (the blood supply to the anterior OpR is provided by the anterior choroidal artery from the internal carotid artery, the blood supply to the posterior part is provided by the middle and posterior cerebral artery, and the blood supply to the lateral part is provided by the lenticulostriate artery from the middle cerebral artery) (26). Sincoff et al. (27) summarized the three surgical approaches to the OpR area: (i) The trans-cortical approach including the superior, middle, inferior temporal gyrus and the occipital cortex. The effect on the OpR depends on the location of the cortical incision and the amount of cortical resection. The inferior temporal gyrus approach typically leads to fewer injuries to the OpR, due to the anatomical course of the OpR. (ii) The infratemporal approach does not affect the OpR, but the bottom of the temporal lobe often needs to be stretched and the zygomatic arch may need to be removed. (iii) The trans-sylvian approach can avoid the OpR and does not damage the functional language area, but does require the operator to have high anatomical knowledge and superb surgical skills. Meyer’s loop mainly projects forward across the superior aspect of the anterior tip of the lateral ventricle’s temporal horn. Therefore, the inferior temporal gyrus approach should be considered to protect the OpR, and damage to the supplying artery should be minimized. In the present study, the 12 patients who were prepared to adopt the preliminary trans-cortical approach were switched to the adjusted inferior temporal gyrus approach according to the reconstructed OpR.

Assessing the visual evoked potential (VEP) remains one of the most common techniques for intraoperative monitoring and protection of the visual pathway. However, many studies have reported that patients’ postoperative visual function was unfavorable despite VEP monitoring during surgery (28). Some reports note that it was not possible to stably monitor VEP during surgery for patients with VA < 0.4 (16). Individual variation in the anatomy of the OpR also increases the risk of damaging the anterior part of the OpR during surgery (29). The application of neuronavigation integrated with DTI can solve this problem. Nine patients in our study with VA < 0.4 on at least one side were well operated on and using multiple ROIs to seed the OpR generated an accurate prediction of OpR anatomy. The application of DTI integrated with neuronavigation can help to precisely locate the lesion and plan the surgical incision and approach. For deep lesions with unclear boundaries, it can also help the operator judge the degree of lesion resection and the relationship between important functional areas and the lesion. Nonetheless, DTI has a significant disadvantage that it is not a “real-time” surgical adjunct. Surgeons must consider changes in the intraoperative anatomy, including midline shift, swelling, relaxation of the cisterns, or anatomical changes during or after tumor resection that may make the DTI inaccurate (30, 31). In our study, 25 patients (89.3%) had a postoperative EOR > 90% and no patient’s VA or VFI were worse 2 months after discharge. Furthermore, VA, VFI, visual function, and QOL scores had a statistically significant difference compared to the time of admission (P < 0.05). Apart from this application and the aforementioned strategy of intraoperative OpR positioning (under the subtitle ‘Intraoperative Neuronavigation and Surgery’), other possible explanations can be given for this result as follows: (i) With a special focus on visual protection, we tried to preserve the OpR as much as possible based on intraoperative rapid pathology, especially for the patients with an OpR morphology score of 3 or 4. This may not be the best choice for patients with higher levels of malignant tumors. (ii) The follow-up was not long enough to provide a reliable and convincing conclusion and make inevitable causal inferences. This makes the results have the potential to be inflated.

Previous studies have proved that tumor size and tumor-related edema are associated with unfavorable visual outcomes in patients with intracranial tumors (32, 33). The Meyer loop is closely related to the anterior tip of the lateral ventricle and each additional 1 mm of damage to the Meyer loop causes an additional 5% loss of the upper quadrant (34). In our study, lesion size, edema, and involvement of the lateral ventricle temporal horn were closely associated with OpR morphological damage. In addition, we performed regression analysis between preoperative and postoperative VA, VFI, visual function, QOL, and other clinical factors. The preoperative multiple linear regression results suggest that larger tumor size is the most important indicator of poor visual outcomes. Advanced age (β = -5.33, 95%CI: -7.01~-3.65, P = 0.004), edema (β = -7.46, 95%CI: -10.83~-4.09, P = 0.037), and involvement of the lateral ventricle temporal horn (β = -7.71, 95%CI: -10.50~-4.92, P = 0.011) affected patients’ preoperative QOL the most.

We did not find significant associations of postoperative VA or VFI with sex, age, tumor size, edema, invasion into the temporal horn, or extent of tumor resection. Preoperative OpR morphology likely played a significant role in the preservation and improvement of postoperative visual function in our study (OR = 0.203, 95% CI: 0.036–0.731, P = 0.033). While the aforementioned factors, including lesion size, edema, and involvement of the lateral ventricle temporal horn, were highly correlated with preoperative OpR morphology, they do not appear to be significantly associated with postoperative OpR morphology. This means that these factors can be partially or entirely resolved during surgery, but can also be due to the small sample size of patients. Severe damage to the OpR, such as deformation or interruption, cannot be reversed. Lastly, involvement of the lateral ventricle temporal horn (OR = 17.542, 95% CI: 2.678–206.021, P = 0.007) was the main clinical factor affecting postoperative QOL.

### Strengths and limitations

Our study supports that DTI integrated with neuronavigation is of value for designing surgical strategies to avoid OpR injury in this context and potentially in other pathologies, such as temporal lobe resection for epilepsy. It is supposed to be the first study introducing the application of DTI tractography integrated with neuronavigation in tumor resection with a focus on visual protection to the knowledge of the authors. We extracted as much patient information as possible to conduct a multivariate regression analysis to identify factors related to preoperative and postoperative VA, VFI, visual function and QOL. These findings are useful for assessing a patient’s condition and predicting the prognosis in clinical practice.

Although all patients in our study had satisfactory visual function and QOL outcomes, several limitations should be recognized. Firstly, the number of patients was insufficient to achieve robust results, and the histological types were heterogeneous across patients. Consequently, the involvement of the subcortical bundles cannot be compared. Secondly, due to limited time, an insufficient number of patients, and heterogeneity of tumor pathology, we were unable to conduct a prospective comparative trial. Though similar studies have been discussed, it is difficult to make inevitable causal inferences (35). Thirdly, the follow-up period (2 months) was relatively short, which may cause heterogeneity in the regression results to some extent. Further research needs to be conducted to ensure the applicability and effectiveness of this technology before implementation in routine clinical practice.

## Conclusion

Despite advances in surgical technique, visual damage remains common following surgeries performed in the optic radiation area. The application of neuronavigation integrated with diffusion tensor imaging tractography can support effective tumor resection with less injury to the OpR, thereby reducing postoperative visual function decline and improving quality of life. Tumor size was identified as the main reason for poor preoperative visual function. Only severe damage to OpR morphology, whether caused by the disease itself or accidental injury during surgery, can lead to an irreversible decline in postoperative visual function. Further research involving more patients is needed to ensure the applicability and effectiveness of this strategy.

## Data availability statement

The original contributions presented in the study are included in the article/**Supplementary Material**. Further inquiries can be directed to the corresponding author.

## Ethics statement

This study was approved by the Nanjing Brain Hospital Ethics Committee and informed written consent was obtained from all patients. Written informed consent to participate in this study was provided by the participants’ legal guardian/next of kin.

## Author contributions

Author contributions to the study and the manuscript preparation. Conception and design, JS, RP, HC, and YZ. Methodology, JS, DL, and RP; Investigation, JS, RP, HC, FB, QZ, and YZ. Formal Analysis, JS and DL. Writing Original Draft, JS, RP, HC, and HT. Writing - Review and Editing, JS, RP, and YZ. Supervision, JS and HT. Funding Acquisition, YZ and HT. All authors contributed to the article and approved the submitted version.

## Funding

This study was supported by the project of Nanjing Medical Youth Talent (No. QRX11010).

## Conflict of interest

The authors declare that the research was conducted in the absence of any commercial or financial relationships that could be construed as a potential conflict of interest.

## Publisher’s note

All claims expressed in this article are solely those of the authors and do not necessarily represent those of their affiliated organizations, or those of the publisher, the editors and the reviewers. Any product that may be evaluated in this article, or claim that may be made by its manufacturer, is not guaranteed or endorsed by the publisher.
