# Supplementary material for: Applications of diffusion tensor imaging integrated with neuronavigation to prevent visual damage during tumor resection in the optic radiation area
Source: Front Oncol. 2022 Aug 16;12:955418. doi: 10.3389/fonc.2022.955418 (PMC9424997; doi:10.3389/fonc.2022.955418)
Supplement: Supplementary file 1 [file DataSheet_1.zip › Supplementary Tables/Supplementary Files/Supplementary Table 4.docx]

**Supplementary Table 4.** The paired Wilcoxon signed-rank test to evaluate the efficacy of surgery by comparing the QOL, VF, VA, and VFI values between admission and 2 months after discharge. (QOL: quality of life, VF: visual function, VA: visual acuity, VFI: visual field index, L: left, R: right)

| **Paired Samples Wilcoxon Signed Rank Test** | | |
| --- | --- | --- |
| Group | V | P value |
| Quality of Life | 66 | 0.0019 |
| Visual Function | 105 | 0.0005 |
| Left-VA | 55 | 0.0021 |
| Right-VA | 45 | 0.0024 |
| Left-VFI | 171 | 0.0000928 |
| Right-VFI | 180 | 0.00028 |
