# Supplementary material for: Applications of diffusion tensor imaging integrated with neuronavigation to prevent visual damage during tumor resection in the optic radiation area
Source: Front Oncol. 2022 Aug 16;12:955418. doi: 10.3389/fonc.2022.955418 (PMC9424997; doi:10.3389/fonc.2022.955418)
Supplement: Supplementary file 1 [file DataSheet_1.zip › Supplementary Tables/Supplementary Files/Supplementary Table 1.docx]

**Supplementary Table 1.** Quality of Life Questionnaire.

|  | **Rating** | | | | **Does Someone Help You?** | |
| --- | --- | --- | --- | --- | --- | --- |
| **Activity** | Not at All | A Little | Qulte a Bit | A Lot | Reply 1 | Reply 2 |
| Self-care |  | | | | | |
| How much problem do you have because of your vision in doing the following activities unaided? |  |  |  |  |  |  |
| Bathing | 1 | 2 | 3 | 4 | No | Yes |
| Eating | 1 | 2 | 3 | 4 | No | Yes |
| Dressing | 1 | 2 | 3 | 4 | No | Yes |
| Toileting | 1 | 2 | 3 | 4 | No | Yes |
| Mobility |  | | | | | |
| How much problem do you have because of your vision in doing the following activities unaided? |  |  |  |  |  |  |
| Walking to neighbors | 1 | 2 | 3 | 4 | No | Yes |
| Walking to shops | 1 | 2 | 3 | 4 | No | Yes |
| Doing your usual household chores | 1 | 2 | 3 | 4 | No | Yes |
| Social |  | | | | | |
| Because of your vision problems, do you feel inclined to participate in the following? |  |  |  |  |  |  |
| Attending social functions like weddings, funerals, festivals | 1 | 2 | 3 | 4 | No | Yes |
| Meeting with friends and relatives | 1 | 2 | 3 | 4 | No | Yes |
| Mental |  | | | | | |
| Because of your vision problems do you feel: |  |  |  |  |  |  |
| A burden on others | 1 | 2 | 3 | 4 | ... | ... |
| Dejected | 1 | 2 | 3 | 4 | ... | ... |
| Loss of confidence in doing usual activities | 1 | 2 | 3 | 4 | ... | ... |
