# Supplementary material for: Applications of diffusion tensor imaging integrated with neuronavigation to prevent visual damage during tumor resection in the optic radiation area
Source: Front Oncol. 2022 Aug 16;12:955418. doi: 10.3389/fonc.2022.955418 (PMC9424997; doi:10.3389/fonc.2022.955418)
Supplement: Supplementary file 1 [file DataSheet_1.zip › Supplementary Tables/Supplementary Files/Supplementary Table 2.docx]

**Supplementary Table 2.** Vision Function Questionnaire.

| **Question NO.** | **Question** | **Rating** | | | |
| --- | --- | --- | --- | --- | --- |
|  |  | Not at All | A Little | Quilt a Lot | A Lot |
| 1 | In general, would you say your vision (with glasses if you wear them) is? | 1 (very good) | 2 (good) | 3 (fair) | 4 (poor) |
| 2 | To what extent does your sight limit you in your daily activities? | 1 | 2 | 3 | 4 |
| 3 | How much problem do you have recognizing people across the street? | 1 | 2 | 3 | 4 |
| 4 | How much problem do you have recognizing the face of a person standing near you? | 1 | 2 | 3 | 4 |
| 5 | How much problem do you have recognizing small or minute objects (such as grains or the lines in your hand)? | 1 | 2 | 3 | 4 |
| 6 | When you are walking along, how much problem do you have noticing objects off the side? | 1 | 2 | 3 | 4 |
| 7a | How much problem do you have adjusting to darkness after being in bright light? | 1 | 2 | 3 | 4 |
| 7b | How much problem do you have adjusting to brightness after being in a dark place? | 1 | 2 | 3 | 4 |
| 8 | How much problem do you have locating something when it is surrounded by a lot of other things (like finding a specific food item on your plate)? | 1 | 2 | 3 | 4 |
| 9 | How much problem do you have in recognizing colors? | 1 | 2 | 3 | 4 |
| 10 | When you reach for an object (eg, to take a glass), how much problem do you have in finding it, because it is further away or closer than you thought? | 1 | 2 | 3 | 4 |
| 11a | How much problem do you have in recognizing a person when you are in bright light? | 1 | 2 | 3 | 4 |
| 11b | How much problem do you have seeing with bright lights shining on your eyes (such as from an oncoming bus or car)? | 1 | 2 | 3 | 4 |
