# Supplementary material for: Applications of diffusion tensor imaging integrated with neuronavigation to prevent visual damage during tumor resection in the optic radiation area
Source: Front Oncol. 2022 Aug 16;12:955418. doi: 10.3389/fonc.2022.955418 (PMC9424997; doi:10.3389/fonc.2022.955418)
Supplement: Supplementary file 1 [file DataSheet_1.zip › Supplementary Tables/Supplementary Files/Supplementary Table 3.docx]

**Supplementary Table 3.** Univariate and multivariate regression analyses of clinical factors for pre/post-operative outcomes from the patients. (OpR: optic radiation, DV: dependent variable, IV: independent variable, VA: visual acuity, VFI: visual field index, VF: visual function, QOL: quality of life, L: left, R: right)

| **Postoperative** | | | | | | | |
| --- | --- | --- | --- | --- | --- | --- | --- |
| **DV: OpR morphology** | | | | | | | |
| **Univariate Analysis** | **IV** | **Estimate/B** | **Std. Error** | **z value** | **Wald** | **P value** | **OR_with_CI** |
|  | Age | -0.399 | 0.233 | -1.71 | 2.934 | 0.099 | 0.671(0.425~1.059) |
|  | Size | -0.475 | 0.0956 | -4.97 | 24.7 | <0.001 | 0.622(0.515~0.75) |
|  | Sex | -0.569 | 0.384 | -1.482 | 2.197 | 0.15 | 0.566(0.267~1.201) |
|  | Edema | -1.5 | 0.2945 | -5.09 | 25.936 | <0.001 | 0.223(0.125~0.397) |
|  | Ventricle | -1.144 | 0.331 | -3.45 | 11.92 | 0.0019 | 0.319(0.167~0.61) |
| **Multivariate Analysis** | **IV** | **Estimate/B** | **Std. Error** | **z value** | **Wald** | **P value** | **OR_with_CI** |
|  | Size | -0.288 | 0.085 | -3.37 | 11.38 | 0.0026 | 0.75(0.634~0.886) |
|  | Edema | -0.721 | 0.28 | -2.57 | 6.62 | 0.017 | 0.486(0.281~0.842) |
|  | Ventricle | -0.596 | 0.233 | -2.56 | 6.53 | 0.0177 | 0.551(0.349~0.87) |
|  | Sex | -0.367 | 0.215 | -1.71 | 2.91 | 0.102 | 0.693(0.455~1.056) |
| **DV: QOL** | | | | | | | |
| **Univariate Analysis** | **IV** | **Estimate/B** | **Std. Error** | **z value** | **Wald** | **P value** | **OR_with_CI** |
|  | Sex | -3.075 | 4.5 | -0.683 | 0.467 | 0.5 | - |
|  | Age | -7.664 | 2.352 | -3.259 | 10.618 | 0.003 | - |
|  | Size | -4.756 | 1.195 | -3.98 | 15.838 | <0.001 | - |
|  | Edema | -14.969 | 3.704 | -4.042 | 16.336 | <0.001 | - |
|  | Ventricle | -12.943 | 3.765 | -3.438 | 11.818 | 0.003 | - |
| **Multivariate Analysis** | **IV** | **Estimate/B** | **Std. Error** | **z value** | **Wald** | **P value** | **OR_with_CI** |
|  | Age | -5.334 | 1.68 | -3.175 | 10.078 | 0.004 | - |
|  | Size | -1.758 | 1.083 | -1.623 | 2.634 | 0.118 | - |
|  | Edema | -7.462 | 3.37 | -2.214 | 4.903 | 0.037 | - |
|  | Ventricle | -7.711 | 2.787 | -2.766 | 7.653 | 0.011 | - |
| **DV: VF** | | | | | | | |
| **Univariate Analysis** | **IV** | **Estimate/B** | **Std. Error** | **z value** | **Wald** | **P value** | **OR_with_CI** |
|  | Sex | -1.232 | 0.852 | -1.446 | 2.091 | 0.148 | - |
|  | Age | -1.241 | 0.724 | -1.716 | 2.944 | 0.086 | - |
|  | Size | -0.788 | 0.354 | -2.227 | 4.96 | 0.026 | - |
|  | Edema | -0.934 | 0.926 | -1.008 | 1.016 | 0.313 | - |
|  | Ventricle | -0.542 | 0.816 | -0.664 | 0.44 | 0.5069 | - |
| **Multivariate Analysis** | **IV** | **Estimate/B** | **Std. Error** | **z value** | **Wald** | **P value** | **OR_with_CI** |
|  | Age | -1.1437 | 0.87 | -1.314 | 1.727 | 0.189 | - |
|  | Size | -0.7357 | 0.375 | -1.961 | 3.846 | 0.049 | - |
| **DV: VA (L)** | | | | | | | |
| **Univariate Analysis** | **IV** | **Estimate/B** | **Std. Error** | **z value** | **Wald** | **P value** | **OR_with_CI** |
|  | Sex | -0.016 | 0.114 | -0.144 | 0.021 | 0.887 | - |
|  | Age | -0.182 | 0.06 | -3.021 | 9.124 | 0.006 | - |
|  | Size | -0.138 | 0.027 | -5.158 | 26.605 | <0.001 | - |
|  | Edema | -0.24 | 0.109 | -2.203 | 4.853 | 0.037 | - |
|  | Ventricle | -0.261 | 0.102 | -2.556 | 6.531 | 0.017 | - |
| **Multivariate Analysis** | **IV** | **Estimate/B** | **Std. Error** | **z value** | **Wald** | **P value** | **OR_with_CI** |
|  | Age | -0.103 | 0.046 | -2.255 | 5.086 | 0.034 | - |
|  | Size | -0.105 | 0.026 | -4.09 | 16.798 | <0.001 | - |
|  | Ventricle | -0.159 | 0.072 | -2.203 | 4.852 | 0.037 | - |
| **DV: VA (R)** | | | | | | | |
| **Univariate Analysis** | **IV** | **Estimate/B** | **Std. Error** | **z value** | **Wald** | **P value** | **OR_with_CI** |
|  | Sex | -0.071 | 0.109 | -0.65 | 0.423 | 0.521 | - |
|  | Age | -0.118 | 0.063 | -1.86 | 3.46 | 0.074 | - |
|  | Size | -0.15 | 0.022 | -6.93 | 48.072 | <0.001 | - |
|  | Edema | -0.284 | 0.099 | -2.85 | 8.154 | 0.008 | - |
|  | Ventricle | -0.185 | 0.104 | -1.79 | 3.219 | 0.084 | - |
| **Multivariate Analysis** | **IV** | **Estimate/B** | **Std. Error** | **z value** | **Wald** | **P value** | **OR_with_CI** |
|  | Size | -0.15 | 0.022 | -6.93 | 48.072 | <0.001 | - |
| **DV: VFI (L)** | | | | | | | |
| **Univariate Analysis** | **IV** | **Estimate/B** | **Std. Error** | **z value** | **Wald** | **P value** | **OR_with_CI** |
|  | Sex | -0.108 | 0.095 | -1.148 | 1.317 | 0.262 | - |
|  | Age | -0.137 | 0.053 | -2.57 | 6.597 | 0.016 | - |
|  | Size | -0.133 | 0.019 | -6.89 | 47.602 | <0.001 | - |
|  | Edema | -0.289 | 0.084 | -3.456 | 11.945 | 0.0019 | - |
|  | Ventricle | -0.152 | 0.092 | -1.643 | 2.698 | 0.113 | - |
| **Multivariate Analysis** | **IV** | **Estimate/B** | **Std. Error** | **z value** | **Wald** | **P value** | **OR_with_CI** |
|  | Age | -0.062 | 0.036 | -1.71 | 2.92 | 0.1 | - |
|  | Size | -0.105 | 0.023 | -4.48 | 20.04 | <0.001 | - |
|  | Edema | -0.093 | 0.069 | -1.35 | 1.83 | 0.188 | - |
| **DV: VFI (R)** | | | | | | | |
| **Univariate Analysis** | **IV** | **Estimate/B** | **Std. Error** | **z value** | **Wald** | **P value** | **OR_with_CI** |
|  | Sex | -0.088 | 0.099 | -0.893 | 0.798 | 0.38 | - |
|  | Age | -0.127 | 0.057 | -2.227 | 4.957 | 0.035 | - |
|  | Size | -0.143 | 0.019 | -7.73 | 59.82 | <0.001 | - |
|  | Edema | -0.303 | 0.087 | -3.503 | 12.273 | 0.002 | - |
|  | Ventricle | -0.138 | 0.097 | -1.415 | 2.003 | 0.169 | - |
| **Multivariate Analysis** | **IV** | **Estimate/B** | **Std. Error** | **z value** | **Wald** | **P value** | **OR_with_CI** |
|  | Size | -0.143 | 0.019 | -7.73 | 59.82 | <0.001 | - |
| **Postoperative** | | | | | | | |
| **DV: QOL** | | | | | | | |
| **Univariate Analysis** | **IV** | **Estimate/B** | **Std. Error** | **z value** | **Wald** | **P value** | **OR_with_CI** |
|  | Sex | -0.065 | 0.776 | -0.083 | 0.007 | 0.934 | 0.938(0.198~4.343) |
|  | Age | 0.782 | 0.553 | 1.414 | 1.999 | 0.157 | 2.185(0.803~7.596) |
|  | Size | 0.486 | 0.289 | 1.68 | 2.819 | 0.093 | 1.626(0.954~3.053) |
|  | Edema | 1.386 | 0.92 | 1.506 | 2.26 | 0.132 | 4(0.743~31.704) |
|  | sight3 | -0.776 | 0.437 | -1.78 | 3.154 | 0.076 | 0.46(0.176~1.022) |
|  | Ventricle | 2.688 | 0.969 | 2.77 | 7.672 | 0.006 | 14.625(2.556~128.043) |
|  | excision1 | -0.414 | 0.505 | -0.821 | 0.674 | 0.412 | 0.661(0.217~1.785) |
| **Multivariate Analysis** | **IV** | **Estimate/B** | **Std. Error** | **z value** | **Wald** | **P value** | **OR_with_CI** |
|  | Age | 1.079 | 0.805 | 1.34 | 1.798 | 0.179 | 2.944(0.748~21.015) |
|  | Ventricle | 2.86 | 1.07 | 2.67 | 7.165 | 0.007 | 17.542(2.678~206.021) |
| **DV: VF** | | | | | | | |
| **Univariate Analysis** | **IV** | **Estimate/B** | **Std. Error** | **z value** | **Wald** | **P value** | **OR_with_CI** |
|  | Sex | 0.2877 | 0.759 | 0.379 | 0.143 | 0.705 | 1.333(0.3~6.1) |
|  | Age | 0.443 | 0.48 | 0.922 | 0.851 | 0.356 | 1.557(0.619~4.258) |
|  | Size | 0.508 | 0.289 | 1.756 | 3.084 | 0.079 | 1.663(0.979~3.123) |
|  | Edema2 | <0.001 | 0.788 | 0 | 0 | 1 | 1(0.208~4.809) |
|  | sight5 | -0.665 | 0.412 | -1.62 | 2.61 | 0.106 | 0.514(0.211~1.103) |
|  | Ventricle | 0.875 | 0.776 | 1.128 | 1.272 | 0.259 | 2.4(0.537~11.697) |
|  | excision1 | 0.375 | 0.52 | 0.72 | 0.518 | 0.472 | 1.455(0.544~4.791) |
| **Multivariate Analysis** | **IV** | **Estimate/B** | **Std. Error** | **z value** | **Wald** | **P value** | **OR_with_CI** |
|  | Edema2 | -2.447 | 1.462 | -1.674 | 2.8 | 0.094 | 0.087(0.003~1.145) |
|  | sight5 | -1.592 | 0.746 | -2.134 | 4.56 | 0.033 | 0.203(0.036~0.731) |
| **DV: VA (L)** | | | | | | | |
| **Univariate Analysis** | **IV** | **Estimate/B** | **Std. Error** | **z value** | **Wald** | **P value** | **OR_with_CI** |
|  | Sex | -0.405 | 0.799 | -0.507 | 0.257 | 0.612 | 0.667(0.13~3.163) |
|  | Age | 0.632 | 0.548 | 1.153 | 1.329 | 0.249 | 1.881(0.692~6.434) |
|  | Size | 0.391 | 0.282 | 1.386 | 1.921 | 0.165 | 1.479(0.871~2.712) |
|  | Edema2 | 1.163 | 0.922 | 1.262 | 1.592 | 0.207 | 3.2(0.589~25.36) |
|  | sight5 | -0.362 | 0.4 | -0.904 | 0.818 | 0.366 | 0.696(0.3~1.501) |
|  | Ventricle | 0.223 | 0.791 | 0.282 | 0.08 | 0.778 | 1.25(0.26~6.066) |
|  | excision1 | 0.261 | 0.556 | 0.47 | 0.221 | 0.638 | 1.299(0.472~4.97) |
| **Multivariate Analysis** | **IV** | **Estimate/B** | **Std. Error** | **z value** | **Wald** | **P value** | **OR_with_CI** |
|  | Age | 0.913 | 0.634 | 1.439 | 2.071 | 0.15 | 2.492(0.792~10.47) |
|  | Edema2 | 1.668 | 1.049 | 1.589 | 2.524 | 0.112 | 5.301(0.775~54.168) |
|  | excision1 | 0.963 | 0.705 | 1.36 | 1.863 | 0.172 | 2.62(0.773~14.184) |
| **DV: VA (R)** | | | | | | | |
| **Univariate Analysis** | **IV** | **Estimate/B** | **Std. Error** | **z value** | **Wald** | **P value** | **OR_with_CI** |
|  | Sex | -0.117 | 0.813 | -0.145 | 0.021 | 0.885 | 0.889(0.171~4.417) |
|  | Age | -0.053 | 0.496 | -0.107 | 0.011 | 0.915 | 0.948(0.36~2.657) |
|  | Size | 1.414 | 0.54 | 2.619 | 6.858 | 0.009 | 4.116(1.751~16.357) |
|  | Edema2 | 0.5 | 0.164 | 3.047 | 9.286 | 0.005 | 1.649(1.195~2.274) |
|  | sight5 | -1.09 | 0.516 | -2.12 | 4.494 | 0.034 | 0.335(0.102~0.827) |
|  | Ventricle | 0.542 | 0.816 | 0.664 | 0.44 | 0.507 | 1.719(0.347~9.033) |
|  | excision1 | -0.639 | 0.524 | -1.22 | 1.488 | 0.223 | 0.528(0.164~1.445) |
| **Multivariate Analysis** | **IV** | **Estimate/B** | **Std. Error** | **z value** | **Wald** | **P value** | **OR_with_CI** |
|  | Age | -2.418 | 1.355 | -1.785 | 3.186 | 0.074 | 0.089(0.002~0.74) |
|  | Size | 2.29 | 0.936 | 2.455 | 6.028 | 0.014 | 9.96(2.559~139.459) |
|  | excision1 | -1.788 | 1.357 | -1.317 | 1.735 | 0.188 | 0.167(0.004~1.104) |
| **DV: VFI (L)** | | | | | | | |
| **Univariate Analysis** | **IV** | **Estimate/B** | **Std. Error** | **z value** | **Wald** | **P value** | **OR_with_CI** |
|  | Sex | -1.54 | 0.852 | -1.808 | 3.268 | 0.071 | 0.214(0.035~1.064) |
|  | Age | -0.352 | 0.511 | -0.689 | 0.475 | 0.491 | 0.703(0.233~1.842) |
|  | Size | 0.373 | 0.29 | 1.287 | 1.657 | 0.198 | 1.453(0.848~2.718) |
|  | Edema2 | 0.955 | 0.823 | 1.161 | 1.349 | 0.245 | 2.6(0.521~13.823) |
|  | sight5 | -0.249 | 0.393 | -0.634 | 0.402 | 0.526 | 0.779(0.345~1.676) |
|  | Ventricle | -0.223 | 0.791 | -0.282 | 0.08 | 0.778 | 0.8(0.165~3.84) |
|  | excision1 | -0.261 | 0.556 | -0.47 | 0.221 | 0.638 | 0.77(0.201~2.116) |
| **Multivariate Analysis** | **IV** | **Estimate/B** | **Std. Error** | **z value** | **Wald** | **P value** | **OR_with_CI** |
|  | Sex | -1.815 | 0.94 | -1.931 | 3.729 | 0.054 | 0.163(0.021~0.911) |
|  | Size | 0.498 | 0.335 | 1.486 | 2.21 | 0.137 | 1.646(0.9~3.47) |
| **DV: VFI (R)** | | | | | | | |
| **Univariate Analysis** | **IV** | **Estimate/B** | **Std. Error** | **z value** | **Wald** | **P value** | **OR_with_CI** |
|  | Sex | 0.677 | 0.793 | 0.854 | 0.73 | 0.393 | 1.969(0.426~10.029) |
|  | Age | 0.209 | 0.474 | 0.442 | 0.195 | 0.659 | 1.233(0.477~3.19) |
|  | Size | 0.009 | 0.259 | 0.034 | 0.001 | 0.973 | 1.009(0.602~1.705) |
|  | Edema2 | 1.361 | 0.832 | 1.634 | 2.671 | 0.102 | 3.9(0.792~21.848) |
|  | sight5 | -0.161 | 0.383 | -0.42 | 0.177 | 0.674 | 0.851(0.389~1.807) |
|  | Ventricle | 0.064 | 0.776 | 0.083 | 0.007 | 0.934 | 1.067(0.23~5.044) |
|  | excision1 | -0.742 | 0.681 | -1.09 | 1.188 | 0.276 | 0.476(0.081~1.452) |
| **Multivariate Analysis** | **IV** | **Estimate/B** | **Std. Error** | **z value** | **Wald** | **P value** | **OR_with_CI** |
|  | Edema2 | 0.745 | 0.66 | 1.129 | 1.275 | 0.259 | 2.107(0.638~8.896) |
|  | sight5 | 2.574 | 1.434 | 1.795 | 3.221 | 0.073 | 13.127(1.06~345.001) |
